# Supplementary material for: Recruitment of cognitive control regions during effortful self-control is associated with altered brain activity in control and reward systems in dieters during subsequent exposure to food commercials
Source: PeerJ. 2019 Feb 28;7:e6550. doi: 10.7717/peerj.6550 (PMC6397754; doi:10.7717/peerj.6550)
Supplement: Supplemental Information 1 [file peerj-07-6550-s001.docx]

**Supplemental Materials**

*Exploratory Analysis Using Larger Set of Nodes in the Frontoparietal Control Network*

In order to select a comprehensive set of regions, we consulted studies that have utilized resting-state functional connectivity data to define stable nodes in the frontoparietal control network with graph theory approaches (i.e. community assignment) (Dosenbach et al., 2007; Rosvall & Bergstrom, 2008; Power et al., 2011). Specifically, we extracted mean activity (for both effortful self-control and cue reactivity tasks) in 31 nodes of the frontoparietal control network, based on a recent parcellation from our group in a large (N=828), independent sample (Huckins et al., 2019). For each node, we used a spherical ROI with a 6-millimeter radius. For the cue reactivity task, we re-computed regulation–reward balance scores by taking the difference between mean activity in this larger definition of the frontoparietal network (with 31 nodes) and activity in reward regions, specifically bilateral ventral striatum (VS) and lateral orbitofrontal cortex (OFC)—using the same regions reported in the main text (MNI coordinates for OFC: -30, 33, -18 ; VS: ± 9, 3, -6; taken from Wagner et al., 2013).

For the effortful self-control (first) task, we observed that across 31 nodes of the frontoparietal control network there was significant activity for the words–versus–film contrast, mean parameter estimate = 0.129 (95% CI: 0.063, 0.195), SD = 0.166, *t*(26) = 4.03, Cohen’s *d* = 0.775, *p* < .001. Relating frontoparietal network activity from the first (effortful self-control) task to balance scores from the second (cue reactivity) task, there was a non-significant relationship in the predicted (negative) direction, *r* = -0.27, *p* = .188. However, there was a significant negative association between self-control related activity from the same DLPFC reported in the main text (MNI Coordinates: 30, 42, 24) and the newly-computed balance scores—with 31 frontoparietal nodes—from the food cue reactivity task, *r* = -0.49, *p* = .012 (95% CI: -0.120, -0.734). This relationship held when controlling for participants’ self-reported difficulty and tiredness when they performed the effortful self-control task, as well as participants’ body mass index (BMI), *b* = -2.034 (95% CI: -3.513, -0.553), *t*(19) = -2.875, *p* = .010.

References

Dosenbach, N. U., Fair, D. A., Miezin, F. M., Cohen, A. L., Wenger, K. K., Dosenbach, R. A., ... & Schlaggar, B. L. (2007). Distinct brain networks for adaptive and stable task control in humans. *Proceedings of the National Academy of Sciences*, *104*(26), 11073-11078.

Huckins, J. F., Adeyemo, B., Miezin, F. M., Power, J. D., Gordon, E. M., Laumann, T. O., ... & Kelley, W. M. (2019). Reward‐related regions form a preferentially coupled system at rest. *Human Brain Mapping*, *40*(2), 361-376.

Power, J. D., Cohen, A. L., Nelson, S. M., Wig, G. S., Barnes, K. A., Church, J. A., ... & Petersen, S. E. (2011). Functional network organization of the human brain. *Neuron*, *72*(4), 665-678.

Rosvall, M., & Bergstrom, C. T. (2008). Maps of random walks on complex networks reveal community structure. *Proceedings of the National Academy of Sciences*, *105*(4), 1118-1123.

Wagner, D. D., Altman, M., Boswell, R. G., Kelley, W. M., & Heatherton, T. F. (2013). Self-regulatory depletion enhances neural responses to rewards and impairs top-down control. *Psychological Science*, *24*(11), 2262-2271.
